# Supplementary material for: Geometry of antiparallel microtubule bundles regulates relative sliding and stalling by PRC1 and Kif4A
Source: eLife. 2018 Oct 24;7:e32595. doi: 10.7554/eLife.32595 (PMC6200392; doi:10.7554/eLife.32595)
Supplement: Figure 8—source data 1. [file elife-32595-fig8-data1.docx]

Figure 8-source data 1

| Concentration | $K_{L}$  (nm) | $v_{0}$  (nm/s) | $a$(%)* | $N(K_{L})$  (# of molecules) | $S$ |
| --- | --- | --- | --- | --- | --- |
| 0.2 nM PRC1+  6 nM Kif4A-GFP | 1597 | 82 | 1 | 2 | 0.3 |
|  |  |  | 5 | 10 | 0.07 |
|  |  |  | 10 | 20 | 0.03 |

*Based on the experimental fluorescence intensity measurements, the occupancy (*a*) is estimated to be 1% if PRC1 molecules can crosslink to all 13 microtubule protofilaments of the moving microtubule and 10% assuming that effective crosslinks are only formed with one protofilament. Considering the molecular structure of PRC1, the values are likely to be in the 1-10% range.
